# Longer-term neurological and endocrine/metabolic conditions in the deepwater horizon oil spill coast guard cohort study: A principal component analysis

**DOI:** 10.1007/s00420-026-02228-0

**Published:** 2026-08-12

**Authors:** Hristina Denic-Roberts, Jordan McAdam, Lawrence S. Engel, Jeanine M. Buchanich, Rachel G. Miller, Evelyn O. Talbott, Dana L. Thomas, Glen A. Cook, Blake Hansen, Jill E. Emerick, Tina Costacou, Jennifer A. Rusiecki

**Affiliations:** 1 Department of Epidemiology, School of Public Health, University of Pittsburgh, Pittsburgh, PA USA; 2 Department of Preventive Medicine and Biostatistics, Uniformed Services University of the Health Sciences, 4301 Jones Bridge Road, Room E-2009, Bethesda, MD USA; 3 Henry M. Jackson Foundation for the Advancement of Military Medicine, Inc., Bethesda, MD USA; 4 Department of Medicine, Uniformed Services University of the Health Sciences, 4301 Jones Bridge Road, Room E-2009, Bethesda, MD USA; 5 Department of Epidemiology, Gillings School of Public Health, University of North Carolina, Chapel Hill, NC USA; 6 Department of Biostatistics, School of Public Health, University of Pittsburgh, Pittsburgh, PA USA; 7 United States Coast Guard Headquarters, Directorate of Health, Safety, and Work Life, Washington, D.C USA; 8 Department of Neurology, Uniformed Services University of the Health Sciences, Bethesda, MD USA; 9 Department of Pediatrics, Uniformed Services University of the Health Sciences, Bethesda, MD USA

**Keywords:** Oil spill, Deepwater Horizon, Neurological health, Endocrine health, Metabolic health, Responder, Crude oil

## Abstract

**Purpose:**

*Deepwater Horizon* oil spill (DWHOS) responders potentially experienced multiple stressors. We examined associations between DWHOS deployment-related stressor patterns and neurological and endocrine/metabolic conditions among U.S. Coast Guard active duty responders who completed post-deployment surveys (n = 3102).

**Methods:**

Deployment-related stressor patterns were identified using principal component analysis of self-reported stressors. Neurological and endocrine/metabolic outcomes were classified from military health encounter records. Associations between principal components (PCs) and incident outcomes (2010–2015) were estimated through Cox proportional hazards regression.

**Results:**

A five-component solution explained 79.2% of the variance. PC1 (high physical/moderate chemical stressor pattern) was associated with *migraine* [adjusted hazard ratio (aHR): 1.25, 95% confidence interval (CI): 1.01–1.56]. PC2 (high crude oil/dispersant stressor pattern) was associated with several migraine/headache conditions including *migraine with aura* (1.54, 1.14–2.07), *mononeuritis of upper limb and mononeuritis multiplex* (1.36, 1.11–1.68), *disorders of lipid metabolism* (1.11, 1.01–1.23*)* and its subcategory *other and unspecified hyperlipidemia* (1.11, 1.01–1.24), and *dysmetabolic syndrome X* (1.74, 1.19–2.54). PC3 (high fatigue/moderate chemical inhalation stressor pattern) was associated with several migraine/headache conditions (aHR range: 1.18–1.60), *disorders of lipid metabolism* (1.11, 1.00–1.23) including *other and unspecified hyperlipidemia* (1.13, 1.01–1.26)*,* and *overweight and obesity* (1.13, 1.00–1.27). PC4 (high anxiety/modest fatigue stressor pattern) was associated with *abnormality of gait* (1.34, 1.03–1.73). PC5 (high back pain) was associated with *mononeuritis of lower limb,* several balance and gait-related conditions*,* thyroid disorders, and *obesity* (aHRs range: 1.16–1.67).

**Conclusion:**

Oil spill response stressor patterns were associated with increased risks of multiple longer-term neurological and endocrine/metabolic conditions, with associations varying by PC.

## Introduction

The semi-submersible offshore oil drilling rig, *Deepwater Horizon* (DWH)*,* exploded on April 20, 2010, and two days later, the rig completely sank approximately 40 miles off the coast of Louisiana, resulting in the largest marine oil spill in U.S. history (Federal On Scene Coordinator 2011; Graham et al. 2011). After 87 days of fresh crude oil flowing, the well was capped on July 15, 2010. The U.S. Coast Guard (USCG) led the national interagency response and deployed nearly 9000 service members to assist in the cleanup (Federal On Scene Coordinator 2011; Graham et al. 2011). The main operational cleanup phase lasted through December 17, 2010. It has been estimated that over the course of the spill, the DWH disaster resulted in the spillage of 185–210 million gallons of crude oil into coastal waters (Berenshtein et al. 2020; Crone & Tolstoy 2010; Federal On Scene Coordinator 2011; Graham et al. 2011; McNutt et al. 2012). Additionally, an unprecedented amount (approximately 1.8 million gallons) of oil dispersants (i.e., COREXIT 9527A and 9500A) was applied on the water’s surface and subsea to disperse the spilled oil (Federal On Scene Coordinator 2011). To burn off spilled oil from the water surface, 411 controlled in situ burns were employed (Federal On Scene Coordinator 2011).

DWH spill responders were potentially exposed to various health hazards. Beyond the chemical exposures to crude oil, oil dispersants, and particulate matter from the controlled in situ burns and flaring of oil and gas from the seabed to the water’s surface, spill responders also experienced other types of stressors. The National Institute for Occupational Safety and Health (NIOSH) has identified ambient heat as one of the main exposures of concern for DWH oil spill responders (National Institute for Occupational Safety and Health 2011), given that the summer of 2010 was one of the hottest on record for the southern U.S. Use of personal protective equipment (PPE), such as full-body suits, further contributed to heat stress (Erickson et al. 2019). Along with high heat, DWH oil spill responders may have also experienced other physical stressors, such as work-related injuries (e.g., falls, strains) (Keshav et al. 2025), ergonomic stress (e.g., back pain), and fatigue (Institute of Medicine 2010; Rusiecki et al. 2017). On top of chemical and physical stressors, many responders also experienced various psychological stressors including anxiety (Wang et al. 2022), depression (Wang et al. 2022), and loss of sleep. All of these stressors together (i.e., chemical, physical, and psychological) may have adversely affected the health of the DWH cleanup responders.

To date, studies of health effects in the oil spill literature have largely focused on examining associations between oil spill response exposures and health outcomes focusing on one or two exposures at a time, and primarily investigating chemical exposures. While the urgency of examining acute and longer-term health effects in relation to specific exposures such as crude oil and oil dispersant exposures remains, it is also important to consider a more realistic responder exposure scenario by studying multiple stressors simultaneously. However, to our knowledge, only a handful of studies to date have focused on combinations of stressors; only three of them investigated associations between the combination of exposures and health outcomes. Peres and colleagues studied associations between multiple survey-based DWH oil spill binary exposure indicators and acute health outcomes among women who resided in the proximity of the DWH oil spill (Peres et al. 2016). Using an exposure mixtures approach, exploratory factor analysis, those investigators identified two factors, physical-environmental exposure and economic exposure, and observed associations between both factors and increased prevalence of multiple acute outcomes, including neurological symptoms (Peres et al. 2016). In a study among oil spill workers, the Gulf Long-term Follow-up Study (GuLF Study), investigators applied an exposure mixtures approach to study associations between highly correlated blood chemical levels of benzene, toluene, ethylbenzene, and xylenes (i.e., BTEX) and neurological symptoms using weighted quantile sum (WQS) regression. A quartile increase in the BTEX exposure mixture, in which benzene was the most heavily weighted component, was associated with increased reporting of both central nervous system (e.g., headache, dizziness) and peripheral nervous system (PNS) (e.g., numbness and tingling in the extremities) symptoms. Gribble and colleagues conducted latent class analysis (LCA) to classify self-reported binary exposures into four distinct exposure profiles among the USCG responders to the DWH oil spill: “low overall exposure,” “low crude oil/exhaust and moderate time outdoors/anxiety,” “high crude oil/exhaust and moderate time outdoors/anxiety,” and “high overall exposure” (Gribble et al. 2022). These patterns of exposure aid in distinguishing similar/less similar responders across a variety of measured and unmeasured exposures. The LCA exposure profiles later were included in an analysis of acute injury risk factors in responders (Keshav et al. 2025). The latent classes of increasingly hazardous exposure were positively associated with slips, trips, and falls; penetrating injuries; and acute symptoms across several organ systems. Additionally, a recent analysis utilized these LCA exposure profiles to evaluate associations between patterns of exposure and self-reported acute symptoms in responders (Horch et al. 2026).

Given the paucity of research examining health effects in association with a realistic scenario of multiple oil spill exposures, the aim of our study was to investigate the risk of longer-term health effects in relation to the DWH cleanup stressor patterns using a data reduction approach suitable for ordinal categorical exposure data, principal component analysis (PCA). Our study population included active duty USCG DWH responders with post-deployment survey data, originating from the well-established Deepwater Horizon Coast Guard (DWH-CG) Cohort study (Rusiecki et al. 2017). Crude oil and oil dispersant constituents have been associated with adverse neurological effects, such as headaches and cognitive impairment, as well as endocrine effects. Expanding upon our previous work of examining associations between self-reported chemical stressors (i.e., exposures to crude oil and to a combination of crude oil and dispersants) and health encounter-based longer-term neurological and endocrine/metabolic outcomes, we first characterized spill response stressor patterns via PCA using self-reported chemical, physical, and psychological stressors and then examined associations between the PCA-identified stressor patterns and risks of longer-term neurological and endocrine/metabolic conditions up to five and a half years following the spill response.

## Methods

### Study population and study design

This study population originated from the DWH-CG Cohort, which has been described previously (Rusiecki et al. 2017). The cohort included 8696 USCG active duty or Selected Reserve service members who were deployed to the DWH oil spill response. Briefly, we excluded 2732 (31%) Selected Reserve responders because only active duty military service members have comprehensive medical coverage through the Military Health System (MHS) and could be followed up continuously for longer-term neurological and endocrine/metabolic outcomes (Rusiecki et al. 2021). We further excluded 2472 of 5964 (41%) active duty responders without any post-deployment survey data. Active duty responders who did not complete post-deployment survey 2 had similar characteristics to active duty responders that completed post-deployment survey 2 (Supplemental Table 1). Active duty responders who completed survey 2 (vs. active duty responders who did not complete survey 2) were more likely to be older (Age 35–50: 28.92 vs. 24.53%), slightly more likely to be female (13.15% vs. 11.11%), and were more likely to be a junior officer (23.86% vs. 18.76%). Race and ethnicity group as well as educational attainment were roughly equal between the groups (~ 78% White, ~ 15% with a Bachelor’s degree). Additionally, we excluded 390 (11%) responders who completed only the first post-deployment survey, which did not acquire data regarding some of the exposures of interest to the present study (e.g., oil dispersants). Those 390 responders were on average deployed two weeks shorter than the rest of the survey takers. Thus, our final study population consisted of 3102 active duty responders who completed the second post-deployment survey that assessed response-related exposures in greater detail.

**Table 1:** Oil spill response stressors selected for PCA among active duty responders from the DWH-CG Cohort (N = 3102)

Self-reported stressor	n (%)
Chemical stressors	
*Crude oil inhalation*	
Never	1464 (47.2%)
Rarely	700 (22.6%)
Sometimes	568 (18.3%)
Most of the time	245 (7.9%)
All of the time	125 (4.0%)
*Direct skin contact with crude oil*	
Never	1985 (64.0%)
Rarely	684 (22.1%)
Sometimes	330 (10.6%)
Most of the time	72 (2.3%)
All of the time	31 (1.0%)
*Oil dispersant contact*	
Never	2634 (84.9%)
Rarely	295 (9.5%)
Sometimes	148 (4.8%)
Most of the time	19 (0.6%)
All of the time	6 (0.2%)
*Exhaust fumes inhalation*	
Never	740 (23.9%)
Rarely	782 (25.2%)
Sometimes	1024 (33.0%)
Most of the time	367 (11.8%)
All of the time	189 (6.1%)
Physical stressors	
*Working in outdoor environment*	
Never	439 (14.2%)
Rarely	436 (14.1%)
Sometimes	582 (18.8%)
Most of the time	691 (22.3%)
All of the time	954 (30.8%)
*Number of PPE items*^1^* used*	
0	595 (19.2%)
1–3	708 (22.8%)
4–6	1093 (35.2%)
7–12	706 (22.8%)
*Back pain*	
Never	2,333 (75.2%)
Sometimes	656 (21.2%)
Most of the time	113 (3.6%)
*Fatigue*	
Never	1,339 (43.2%)
Sometimes	1298 (41.8%)
Most of the time	465 (15.0%)
*Psychological stressor*	
Anxiety	
Never	2711 (87.4%)
Sometimes	352 (11.3%)
Most of the time	39 (1.3%)

Physical stressors	
*Working in outdoor environment*	
Never	439 (14.2%)
Rarely	436 (14.1%)
Sometimes	582 (18.8%)
Most of the time	691 (22.3%)
All of the time	954 (30.8%)
*Number of PPE items*^1^* used*	
0	595 (19.2%)
1–3	708 (22.8%)
4–6	1093 (35.2%)
7–12	706 (22.8%)
*Back pain*	
Never	2,333 (75.2%)
Sometimes	656 (21.2%)
Most of the time	113 (3.6%)
*Fatigue*	
Never	1,339 (43.2%)
Sometimes	1298 (41.8%)
Most of the time	465 (15.0%)
*Psychological stressor*	
Anxiety	
Never	2711 (87.4%)
Sometimes	352 (11.3%)
Most of the time	39 (1.3%)

^1^Waders, safety boots, nitrile/latex gloves, Camelbak, leather work gloves, personal flotation device, respirator, Tyvex suit, hand sanitizer, protective headgear, safety glasses, decontamination stations

Note: The percentages may not add to 100% due to rounding

Abbreviations: PPE, personal protective equipment

#### Statement of ethics approval

This study was approved by the Institutional Review Boards (IRB) of the Uniformed Services University (USU) (FWA 00001628; DoD Assurance P60001), the USCG, and the University of North Carolina, Chapel Hill. A waiver for informed consent was approved by the USU IRB. This study received approval of Protocol G187P9 for Human Subjects Participation.

### Exposure ascertainment

For this study, we considered three different types of deployment-related stressors: chemical, physical, and psychological (Institute of Medicine 2010). The majority of the stressors were ascertained from the second post-deployment survey, which was launched on November 1, 2010. The median response time of survey completion was 153 days after the end of deployment (Rusiecki et al. 2017). Self-reported chemical stressors experienced during the spill response were assessed on a 5-point Likert scale (“never,” “rarely,” “sometimes,” “most of the time,” and “all of the time”) and included: crude oil exposure via different routes (i.e., inhalation, direct skin contact, swallowing/ingestion, submersion of a body part, and being in the vicinity of burning crude oil), contact with oil dispersants, and engine exhaust fumes inhalation. Deployment-related physical stressors included working in an outdoor environment (ascertained on the 5-point Likert scale), use and type of PPE, experience of back pain, fatigue, physical injuries, and ambient heat. We quantified PPE use intensity by categorizing the reported number of PPE items and behaviors (i.e., waders, safety boots, nitrile/latex gloves, Camelbak, leather work gloves, personal flotation device, respirator, Tyvex suit, hand sanitizer, protective headgear, safety glasses, decontamination stations) into four groups (i.e., 0, 1–3, 4–6, and 7–12). Responder responses to physical injury questions were categorized into “none,” “mild” (i.e., scrapes, abrasions, sprains), and “moderate/severe” (i.e., burns, lacerations, punctures, fractures). Ambient heat measurements, described previously, were derived from hourly heat index temperatures relevant to a responder’s deployment period, combined with self-reported time spent outdoors. Spill response-related psychological stressors included survey-reported experience of back pain, fatigue, anxiety, sleep problems, and depression, all measured on a 3-point Likert scale of “never,” “sometimes,” and “most of the time.”

### Outcome ascertainment

Health encounters that included neurological, endocrine, and metabolic conditions were queried from the MHS Data Repository (MDR), a medical health encounter data repository maintained by the U.S. military. This comprehensive data repository contains information from inpatient and outpatient health encounters occurring in both military treatment facilities and clinics (“direct care”) and civilian treatment facilities for which care is billed to the military (“purchased care”). We created a database with comprehensive coverage for all of our active duty cohort members by combining MDR-based health encounters from four major sources: 1) inpatient direct/military care, 2) outpatient direct/military care, 3) inpatient purchased/civilian care, and 4) outpatient purchased/civilian care. We included health encounter records from October 1, 2007 to September 30, 2015, and therefore, had full medical encounter information from approximately two and a half years before the DWH spill, to account for prevalent conditions, until about five and a half years post-spill.

During our study time period, all of the health encounter MDR diagnoses were coded using the Ninth Revision of the *International Classification of Diseases* (ICD-9) codes. We evaluated diagnoses of chronic neurological, endocrine, and metabolic diseases and symptoms classified by three-, four-, or five-digit ICD-9 codes. We assessed individual and grouped ICD-9 codes for various conditions. Full listings of the individual and grouped conditions we evaluated, along with their corresponding ICD-9 codes, are provided in Supplemental Tables 2 (neurologic) and 3 (endocrine and metabolic).

**Table 2:** Characteristics of active duty responders from the DWH-CG Cohort (N = 3102)

Characteristic	
*Age (years)*	
Mean (SD)	31.1 (7.6)
*Sex, n (%)*	
Male	2694 (86.8%)
Female	408 (13.2%)
*Race, n (%)*	
White	2422 (78.1%)
Black	135 (4.3%)
Asian/AI/AN/NH/PI	132 (4.3%)
Other	156 (5.0%)
Unknown	257 (8.3%)
*Military rank/grade, n (%)*	
Junior enlisted (E1-E5)	1453 (46.8%)
Senior enlisted (E6-E10)	799 (25.8%)
Officer (O1-O10, W2-W4)	850 (27.4%)
*Highest education, n (%)*	
High school or less	1971 (63.5%)
Some college or higher*	1094 (35.3%)
Other or not indicated	37 (1.2%)
*Cigarette smoking status, n (%)*	
Never	1888 (60.9%)
Former	521 (16.8%)
Current	693 (22.3%)
*Home station location, n (%)*	
Gulf Coast	902 (29.1%)
Non-Gulf Coast	2200 (70.9%)
*Deployment length (days)*	
Mean (SD)	49.8 (63.8)
*End of deployment*	
Median calendar date	August 6, 2010
*Follow-up time (years)*	
Median	5.05

^*^Some college or higher includes technical school, bachelors, masters, and doctoral degree

Abbreviations: AI, American Indian; AN, Alaska Native; NH, Native Hawaiian; PI, Pacific Islander; SD,  standard deviation

Our incident case definition for classifying neurological, endocrine, and metabolic outcomes required having at least one inpatient or two outpatient encounters/medical visits with a specific individual neurological/endocrine/metabolic condition or a group of conditions in any diagnostic position. Prevalent cases, defined as those who had a pre-existing neurological/endocrine/metabolic condition documented in the MDR before the end of spill deployment (October 1, 2007 – end of deployment) using the same case definition as a post-deployment incident case, were excluded from each corresponding analysis of the particular neurological/endocrine/metabolic outcome. To avoid data sparsity issues, we only retained outcomes for which there were at least 10 incident cases.

### Calculation of person-time

Each responder’s follow-up time started on the day after the last day of their DWH deployment. The end of follow-up time was the earliest of three dates: 1) the date of becoming an incident case of a particular neurological/endocrine/metabolic condition, 2) the end of the follow-up period (September 30, 2015), or 3) the USCG exit date.

### Statistical analyses

#### Characterization of stressor patterns

To identify common DWH response stressor patterns based on chemical, physical, and psychological stressors, we conducted a PCA. This commonly used dimensionality reduction technique aims to explain as much of the total variance in the data as possible using a reduced number of variables by creating new, uncorrelated variables (i.e., principal component scores) that represent weighted linear combinations of the original input variables. Principal components (PCs) aim to characterize an underlying, unobserved structure in the data. We performed PCA with varimax (orthogonal) rotation using nine, standardized ordinal variables in order to identify a reduced number of uncorrelated PCs that represent stressor patterns of the USCG responders to the DWH oil spill. Although we originally considered including a total of 16 variables from chemical, physical, and psychological domains, we selected nine variables across the three domains based on prevalence of the stressors and interpretability of PCs. These 9 variables are listed and described in Table 1. For the final PC solution, we retained a number of PCs based on an a priori set criterion of ~ 80% of total variance being explained by all PCs and interpretability/coherence of identified PCs. The PC interpretability was guided by the pattern of the rotated factor loadings, i.e., weights that each input variable contributes to the PCs. Factor loadings with a value of 0.30 or greater were considered to be significant.

#### Associations between PCs and health outcomes

We used multivariable Cox proportional hazards regression to model associations between the PC scores and time to neurological/endocrine/metabolic incident events by calculating adjusted hazard ratios (aHRs), 95% confidence intervals (CIs), and *p*-values. Each model was co-adjusted for all selected PC scores as continuous exposures and was further adjusted for age at baseline (years), sex (male, female), race (white, Black, other/unknown), and baseline cigarette smoking status (never, former, current). Information on age, sex, and race was obtained from USCG administrative databases. Smoking status was ascertained via post-deployment questionnaire. To account for multiple comparisons, false discovery rate (FDR)-adjusted *p*-values were calculated for each model. The assumption of proportionality of hazards was tested across the overall follow-up period (end of deployment—September 30, 2015) by calculating Pearson correlations between Schoenfeld residuals for each PC score and follow-up time. A *p*-value of < 0.05 for the corresponding Pearson correlation coefficient suggested non-proportionality of hazards and, therefore, a violation of the proportionality assumption. When the assumption was violated, we calculated aHRs and 95% CIs for two approximately equal-length sub-periods: 1) end of deployment (median date August 6, 2010) through December 31, 2012 (hereafter referred to as “the earlier period”) and 2) January 1, 2013 through September 30, 2015 (hereafter referred to as “the later period”).

All analyses were performed in SAS Version 9.4 (SAS Institute, Cary, NC, USA). We used the SAS procedure PROC FACTOR for conducting the PCA.

## Results

Table 2 summarizes the characteristics of our active duty responder study population. The mean age at baseline was 31 years. Responders were predominantly male (86.8%), white (78.1%), and enlisted (i.e., 46.8% junior enlisted; 25.8% senior enlisted). The majority (63.5%) had been educated through high school. More than half of responders (60.9%) reported never smoking cigarettes; 16.8% were former smokers, while 22.3% reported smoking during deployment (current smokers). Approximately one third (29.1%) responded to the DWH spill from a home location along the Gulf Coast. Responders deployed to the DWH spill for an average of 50 days and August 6, 2010 marked the median deployment end date. The median follow-up time lasted 5 years.

### PC solution

**Fig. 1:**
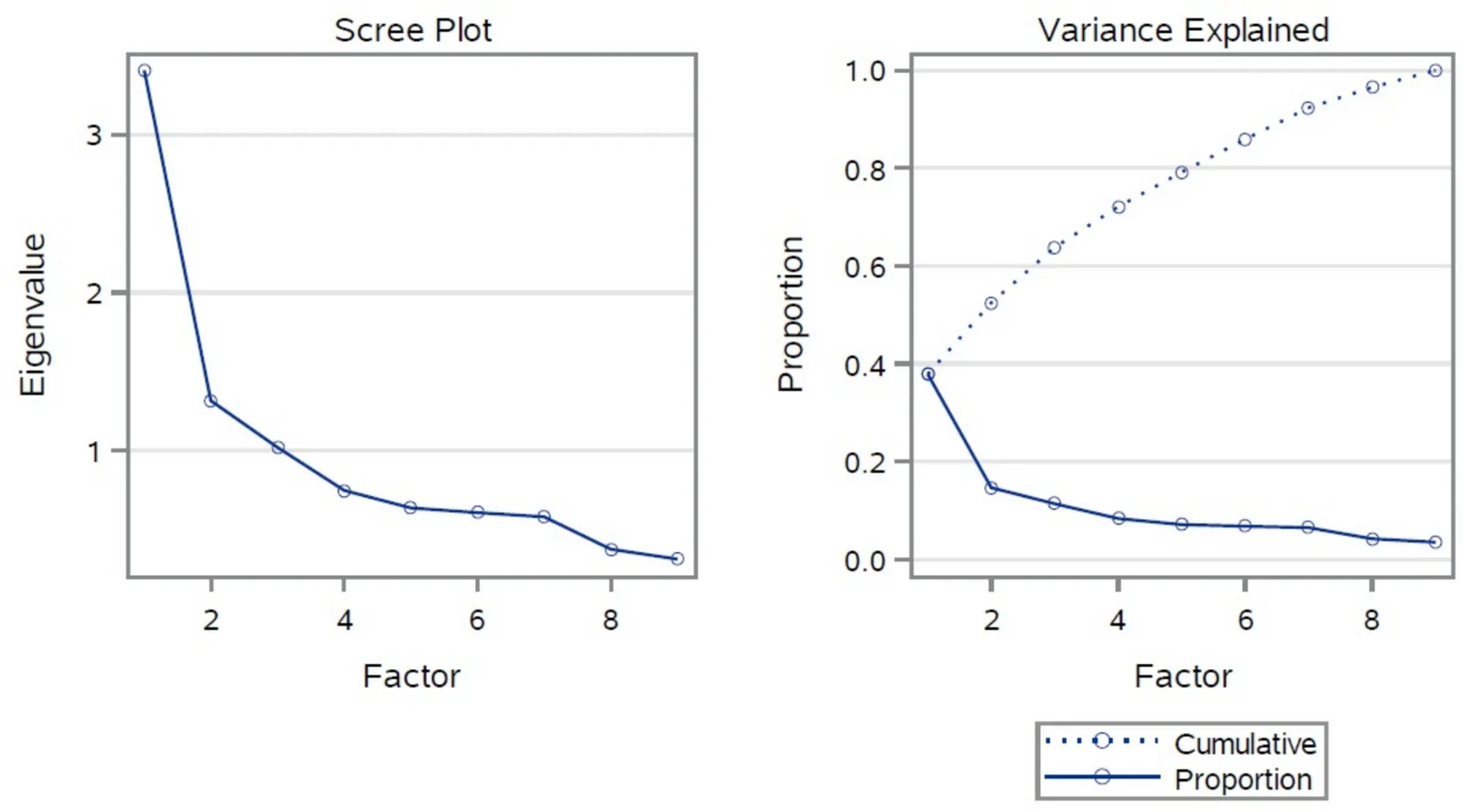
Scree plot and proportion of variance explained plots for the principal component analysis

Frequencies for the seven (out of the original 16 considered) ordinal variables not included in the PCA are presented in Supplemental Table 4. The frequencies of the nine input, survey-based ordinal variables that were used in the PCA are presented in Table 1. These variables included four chemical stressors (i.e., exposures to: crude oil via inhalation and via direct skin contact, oil dispersants, and exhaust fumes inhalation), four physical stressors (i.e., frequency of working outdoors, intensity of PPE use, back pain, and fatigue), and one psychological stressor (i.e., anxiety). The nine input variables were standardized before carrying out the PCA with varimax rotation. PCA identified a five-component solution that accounted for 79.2% of the total variance (Fig. 1).

The five components accounted for 37.9%, 14.6%, 11.3%, 8.3%, and 7.1% of the variance, respectively. The rotated factor loadings for each of the nine standardized variables across each PC are presented in Table 3. The first PC was characterized by high factor loadings for working outdoors and PPE intensity, a moderate loading for exhaust fumes inhalation, and a modest loading for crude oil exposure via direct skin contact (hereafter referred to as PC1 or “High Physical/Moderate Chemical”). The second PC was dominated by high loadings for crude oil exposure via both inhalation and skin contact and oil dispersant exposure (hereafter referred to as PC2 or “High Crude Oil/Oil Dispersant”). The third PC was characterized by a high loading for fatigue, a moderate loading for exhaust fumes inhalation, and a modest loading for crude oil inhalation (hereafter referred to as PC3 or “High Fatigue/Moderate Chemical Inhalation”). The fourth PC was dominated by a high factor loading for anxiety and a modest factor loading for fatigue (hereafter referred to as PC4 or “High Anxiety/Modest Fatigue”). Finally, the fifth PC was dominated by a high factor loading for back pain (hereafter referred to as PC5 or “High Back Pain”).

**Table 3:** Rotated factor loadings for each self-reported DWH oil spill response stressor for the five-component solution

	PC (% variance explained)				
	PC 1 (37.9%)	PC 2 (14.6%)	PC 3 (11.3%)	PC 4 (8.3%)	PC 5 (7.1%)
Self-reported stressor	Rotated factor loadings				
Crude oil inhalation	0.29	**0.72**	**0.32**	− 0.01	0.09
Crude oil direct skin contact	**0.35**	**0.74**	0.10	0.12	0.10
Oil dispersant contact	0.01	**0.84**	0.02	0.01	0.05
Exhaust fumes inhalation	**0.52**	0.14	**0.65**	− 0.12	0.12
PPE intensity	**0.84**	0.22	0.02	0.12	0.09
Working outdoors	**0.86**	0.16	0.16	− 0.06	0.02
Back pain	0.09	0.13	0.19	0.11	**0.96**
Fatigue	− 0.003	0.17	**0.80**	**0.30**	0.17
Anxiety	0.03	0.06	0.14	**0.95**	0.10

In bold: factor loading value ≥ 0.30

Abbreviations: PC, principal component; PPE, personal protective equipment

### Associations between principal components and neurological conditions

In Table 4, we present associations between the five selected PCs and incident neurological conditions following the DWH oil spill response adjusted for age, sex, race, and cigarette smoking and co-adjusted for all PCs. In the overall follow-up period, PC1 was associated with an elevated risk for *migraine* (aHR = 1.25, 95% CI 1.01–1.56). After FDR-adjustment, this finding was not statistically significant. PC1 was also suggestively associated with increased risks for other outcomes related to headaches and migraines, including *migraine with aura* (aHR = 1.48, 95% CI 0.91–2.41), the grouped conditions of *migraine excluding menstrual migraine and persistent migraine aura with cerebral infarction* (aHR = 1.25, 95% CI 0.99–1.56), *headaches/migraines combined* (aHR = 1.14, 95% CI 0.99–1.31), and *headaches/migraines combined excluding menstrual migraine and persistent migraine aura with cerebral infarction* (aHR = 1.14, 95% CI 0.99–1.31).

**Table 4:** Associations between PCs and risk of neurological conditions among active duty DWH-CG Cohort responders (N = 3,102), 2010–2015

		Exposure pattern	PC 1		PC 2		PC 3		PC 4		PC 5	
			High physical/moderate chemical		High crude oil/oil dispersant		High fatigue/moderate chemical inhalation		High anxiety/modest fatigue		High back pain	
Condition (ICD-9 code)	N	Person years	aHR (95% CI)	*p*	aHR (95% CI)	*p*	aHR (95% CI)	*p*	aHR (95% CI)	*p*	aHR (95% CI)	*p*
Migraine (346)	94	13,512	1.25 (1.01–1.56)	0.04	1.12 (0.93–1.35)	0.23	1.29 (1.06–1.58)	0.01	1.00 (0.83–1.20)	1.00	1.15 (0.97–1.37)	0.11
Migraine with aura (346.0)	22	13,978	1.48 (0.91–2.41)	0.11	**1.54 (1.14–2.07)**	** < 0.01**	1.12 (0.73–1.70)	0.60	1.15 (0.84–1.58)	0.39	1.27 (0.90–1.79)	0.17
Migraine, unspecified (346.9)	80	13,978	1.19 (0.95–1.50)	0.14	1.01 (0.81–1.27)	0.93	1.30 (1.05–1.61)	0.02	0.98 (0.80–1.21)	0.85	1.03 (0.83–1.26)	0.78
Migraine excl. menstrual and persistent migraine aura with cerebral infarction (346.1–346.3, 346.5, 346.7–346.9)	88	13,551	1.25 (0.99–1.56)	0.05	1.10 (0.90–1.34)	0.35	1.30 (1.06–1.59)	0.01	1.01 (0.83–1.22)	0.92	1.12 (0.93–1.35)	0.23
Headache (784.0)	145	13,299	1.14 (0.96–1.35)	0.13	1.14 (0.99–1.32)	0.07	1.10 (0.94–1.29)	0.24	1.06 (0.91–1.23)	0.45	1.13 (0.97–1.31)	0.11
Other headache syndromes (339)	44	13,905	1.21 (0.87–1.67)	0.25	1.08 (0.81–1.44)	0.60	**1.60 (1.21–2.11)**	** < 0.01**	0.82 (0.59–1.14)	0.24	1.03 (0.79–1.34)	0.83
Headaches/migraines combined (339, 346, 784.0)	224	12,848	1.14 (0.99–1.31)	0.07	1.15 (1.02–1.29)	0.02	1.18 (1.04–1.35)	0.01	1.06 (0.95–1.19)	0.31	1.09 (0.97–1.23)	0.15
Headaches/migraines combined excl. menstrual and persistent migraine aura with cerebral infarction (339, 346.1–346.3, 346.5, 346.7–346.9, 784.0)	222	12,873	1.14 (0.99–1.31)	0.07	1.15 (1.02–1.29)	0.02	1.18 (1.04–1.34)	0.01	1.07 (0.95–1.20)	0.26	1.08 (0.95–1.22)	0.23
Mononeuritis of upper limb and mononeuritis multiplex (354)	61	13,830	1.10 (0.84–1.44)	0.49	**1.36 (1.11–1.68)**	** < 0.01**	1.20 (0.93–1.55)	0.16	0.96 (0.76–1.22)	0.74	1.16 (0.93–1.44)	0.18
Carpal tunnel syndrome (354.0)	38	13,971	0.95 (0.68–1.33)	0.76	1.25 (0.94–1.66)	0.12	1.21 (0.88–1.66)	0.24	0.76 (0.52–1.12)	0.16	1.25 (0.96–1.63)	0.10
Mononeuritis of lower limb (355)	42	13,944	0.90 (0.66–1.23)	0.51	1.15 (0.87–1.52)	0.33	1.17 (0.86–1.59)	0.32	0.95 (0.70–1.28)	0.74	**1.42 (1.12–1.81)**	** < 0.01**
Visual disturbances (368)	47	13,824	1.05 (0.78–1.41)	0.75	1.00 (0.76–1.33)	1.00	1.24 (0.94–1.63)	0.13	1.14 (0.89–1.45)	0.29	1.09 (0.83–1.42)	0.53
Hearing loss (389)	154	13,432	0.98 (0.83–1.15)	0.81	1.04 (0.88–1.22)*	0.64	1.01 (0.86–1.19)	0.90	0.97 (0.83–1.14)	0.71	1.05 (0.90–1.23)	0.54
Tinnitus (388.3)	74	13,860	1.08 (0.85–1.37)	0.53	1.12 (0.90–1.39)	0.31	1.18 (0.94–1.48)	0.15	1.00 (0.80–1.24)	1.00	1.06 (0.86–1.31)	0.59
Syncope and collapse (780.2)	37	13,877	0.83 (0.60–1.15)	0.26	0.99 (0.71–1.40)	0.95	1.16 (0.84–1.61)	0.37	1.01 (0.75–1.37)	0.95	1.31 (1.00–1.72)	0.05
Dizziness and giddiness (780.4)	76	13,721	1.06 (0.85–1.34)	0.62	0.78 (0.59–1.04)	0.09	1.05 (0.83–1.31)	0.68	1.09 (0.89–1.34)	0.41	1.22 (1.00–1.48)	0.05
Abnormality of gait (781.2)	35	13,911	0.88 (0.63–1.22)	0.45	0.98 (0.71–1.35)	0.90	0.86 (0.61–1.22)	0.39	1.34 (1.03–1.73)	0.03	1.36 (1.03–1.79)	0.03
Disturbance of skin sensation (782.0)	86	13,737	1.17 (0.93–1.46)	0.17	0.88 (0.69–1.11)	0.29	0.97 (0.78–1.21)	0.79	1.08 (0.89–1.31)	0.44	1.19 (0.99–1.43)	0.06

All models are adjusted for age, sex, race, and cigarette smoking, and co-adjusted for all principal components; *Because of the proportionality of hazards assumption violation for PC2 and *hearing loss* during 2010–2015 (Schoenfeld *p* < 0.05), results from sub-period analyses were: 2010–2012: n = 83, aHR = 0.86, 95% CI 0.66–1.12 and 2013–2015: n = 71, aHR = 1.20, 95% CI 0.98–1.46. Bolding denotes statistical significance after false discovery rate (FDR) adjustment (*p* < 0.20)

Abbreviations: aHR, adjusted hazard ratios; CI, confidence interval; DWH-CG, Deepwater Horizon Coast Guard; ICD, International Classification of Disease; PC, principal component

In the overall follow-up period, PC2 was associated with elevated risks for *migraine with aura* (aHR = 1.54, 95% CI 1.14–2.07), *headaches/migraines combined* (aHR = 1.15, 95% CI 1.02–1.29), *headaches/migraines combined excluding menstrual migraine and persistent migraine aura with cerebral infarction* (aHR = 1.15, 95% CI 1.02–1.29), and an inflammatory nerve condition *mononeuritis of upper limb and mononeuritis multiplex* (aHR = 1.36, 95% CI 1.11–1.68). After FDR-adjustment, only *migraine with aura* (*p * < 0.01; *p *_FDR_ < 0.20) and *mononeuritis of upper limb and mononeuritis multiplex* (*p * < 0.01; *p *_FDR_ < 0.20) were still statistically significant. There were also suggestions of an elevated risk for *headache* (aHR = 1.14, 95% CI 0.99–1.32) and a reduced risk for *dizziness and giddiness* (aHR = 0.78, 95% CI 0.59–1.04). The proportionality of hazards assumption was violated for PC2 and one of the outcomes (*hearing loss*), therefore, we conducted the analyses for this outcome separately in the earlier (2010–2012) and in the later time period (2013–2015) (Table 4 footnote). The risk for *hearing loss* was reduced in the earlier time period (aHR = 0.86, 95% CI 0.66–1.12) and elevated in the later time period (aHR = 1.20, 95% CI 0.98–1.46), although neither association reached statistical significance.

PC3 was also associated with increased risks for several conditions related to migraines and headaches in the overall follow-up period, including *migraine* (aHR = 1.29, 95% CI 1.06–1.58) and its subcategory *migraine, unspecified* (aHR = 1.30, 95% CI 1.05–1.61), the grouped outcome *migraine excluding menstrual migraine and persistent migraine aura with cerebral infarction* (aHR = 1.30, 95% CI 1.06–1.59), *other headache syndromes* (aHR = 1.60, 95% CI 1.21–2.11), and the grouped outcomes of *headaches/migraines combined* (aHR = 1.18, 95% CI 1.04–1.35) and *headaches/migraines combined excluding menstrual migraine and persistent migraine aura with cerebral infarction* (aHR = 1.18, 95% CI 1.04–1.34). After FDR-adjustment, only *other headache syndromes* (*p * < 0.01; *p *_FDR_ < 0.20) was still statistically significant.

PC4 was associated with an elevated risk for *abnormality of gait* (aHR = 1.34, 95% CI 1.03–1.73). After FDR-adjustment, this finding was not statistically significant. Lastly, PC5 was also associated with elevated risks for several neurological outcomes, including an inflammatory nerve condition *mononeuritis of lower limb* (aHR = 1.42, 95% CI 1.12–1.81), a condition characterized by fainting *syncope and collapse* (aHR = 1.31, 95% CI 1.00–1.72), *dizziness and giddiness* (aHR = 1.22, 95% CI 1.00–1.48), and *abnormality of gait* (aHR = 1.36, 95% CI 1.03–1.79). After FDR-adjustment, only *mononeuritis of lower limb* (*p *< 0.01; *p *_FDR_ < 0.20) was still statistically significant. There was also a suggestive association between PC5 and risk for *disturbance of skin sensation* (aHR = 1.19, 95% CI 0.99–1.43).

### Associations between PCs and endocrine/metabolic conditions

Multivariable Cox proportional hazards regression associations between the five PCs and incident endocrine and metabolic conditions are reported in Table 5.

**Table 5:** Associations between PCs and risk of endocrine/metabolic conditions among active duty DWH-CG Cohort responders (N = 3,102), 2010–2015

		Exposure pattern	PC 1		PC 2		PC 3		PC 4		PC 5	
			High physical/ moderate chemical		High crude oil/oil dispersant		High fatigue/moderate chemical inhalation		High anxiety/modest fatigue		High back pain	
Condition (ICD-9 code)	N	Person years	aHR (95% CI)	*p*	aHR (95% CI)	*p*	aHR (95% CI)	*p*	aHR (95% CI)	*p*	aHR (95% CI)	*p*
Thyroid disorders combined (240–242,244–246)	54	13,715	1.12 (0.86–1.46)		1.00 (0.75–1.33)*		0.90 (0.67–1.19)		0.97 (0.75–1.25)		1.31 (1.05–1.63)	
Acquired hypothyroidism (244)	35	13,809	1.02 (0.74–1.41)	0.90	0.97 (0.67–1.39)*	0.87	0.81 (0.57–1.16)	0.25	1.05 (0.78–1.41)*	0.75	1.42 (1.10–1.84)	< 0.01
Diabetes mellitus (250)	20	13,969	0.91 (0.59–1.41)	0.67	0.81 (0.47–1.42)	0.46	0.88 (0.55–1.42)	0.60	0.90 (0.53–1.53)*	0.70	1.04 (0.67–1.61)	0.86
Disorders of lipid metabolism (272)	375	11,829	1.02 (0.92–1.14)	0.72	1.11 (1.01–1.23)	0.04	1.11 (1.00–1.23)	0.05	1.05 (0.96–1.15)	0.29	1.02 (0.92–1.12)	0.69
Pure hypercholesterolemia (272.0)	75	13,769	1.23 (0.96–1.57)	0.10	1.20 (0.99–1.46)	0.07	0.86 (0.68–1.10)	0.22	0.96 (0.75–1.21)	0.74	1.05 (0.84–1.30)	0.66
Pure hyperglyceridemia (272.1)	42	13,854	1.24 (0.89–1.75)*	0.21	1.01 (0.75–1.36)	0.95	1.15 (0.85–1.56)	0.37	0.99 (0.74–1.32)	0.95	1.18 (0.91–1.54)	0.22
Mixed hyperlipidemia (272.2)	33	13,899	1.08 (0.75–1.56)	0.68	1.11 (0.81–1.54)	0.52	1.09 (0.77–1.54)	0.63	0.77 (0.49–1.20)	0.25	1.10 (0.80–1.51)	0.56
Other and unspecified hyperlipidemia (272.4)	329	12,170	1.01 (0.90–1.13)	0.86	1.11 (1.01–1.24)	0.05	1.13 (1.01–1.26)	0.03	1.05 (0.95–1.16)	0.34	1.03 (0.93–1.14)	0.57
Gout (274)	30	12,170	1.13 (0.77–1.66)	0.53	1.03 (0.70–1.49)	0.88	1.26 (0.88–1.81)	0.21	0.96 (0.67–1.38)	0.82	1.18 (0.86–1.63)*	0.31
Dysmetabolic syndrome X (277.7)	13	13,969	0.89 (0.49–1.63)	0.70	**1.74 (1.19–2.54)**	** < 0.01**	0.66 (0.33–1.32)	0.24	0.41 (0.10–1.69)	0.22	0.57 (0.26–1.26)	0.16
Overweight, obesity and other hyperalimentation (278)	259	12,822	1.13 (0.99–1.29)	0.07	1.10 (0.99–1.24)	0.10	1.12 (0.99–1.26)	0.07	1.01 (0.90–1.13)	0.86	1.06 (0.94–1.19)	0.33
Overweight and obesity (278.0)	258	12,825	1.13 (0.99–1.29)	0.07	1.11 (0.99–1.24)	0.07	1.13 (1.00–1.27)	0.05	1.01 (0.90–1.13)	0.86	1.06 (0.94–1.19)	0.33
Obesity, unspecified (278.00)	131	13,526	1.05 (0.87–1.25)	0.60	1.13 (0.97–1.32)	0.12	1.00 (0.84–1.18)	1.00	1.00 (0.85–1.18)	1.00	1.16 (1.00–1.36)	0.06
Overweight (278.02)	160	13,343	1.10 (0.93–1.29)	0.25	1.14 (0.99–1.31)	0.07	1.13 (0.97–1.31)	0.11	1.07 (0.93–1.23)	0.34	1.08 (0.94–1.25)*	0.29
Abnormal weight gain (783.1)	18	13,983	1.17 (0.72–1.91)	0.53	1.01 (0.62–1.65)	0.97	1.12 (0.72–1.74)	0.61	0.58 (0.26–1.28)	0.18	0.69 (0.39–1.22)	0.20
Abnormal glucose tolerance test (790.2)	70	13,771	0.87 (0.69–1.10)	0.24	0.78 (0.56–1.07)	0.13	1.12 (0.88–1.43)	0.36	1.15 (0.95–1.40)	0.16	0.98 (0.78–1.25)	0.87

All models are adjusted for age, sex, race, and cigarette smoking, and co-adjusted for all principal components

^*^The proportionality of hazards assumption was violated during 2010–2015 (Schoenfeld *p* < 0.05); results from sub-period analyses (2010–2012 and 2013–2015) are presented in Supplemental Table 5. Bolding denotes statistical significance after false discovery rate (FDR) adjustment (*p* < 0.20)

Abbreviations: aHR, adjusted hazard ratio; CI, confidence interval; DWH-CG, Deepwater Horizon Coast Guard; ICD, International Classification of Disease; PC, principal component

In the overall follow-up period, where the proportionality of hazards assumption was not violated, PC1 was not significantly associated with any of the endocrine and metabolic conditions we evaluated. However, there were suggestions of elevated risks for *overweight, obesity, and other hyperalimentation* and its subcategory *overweight and obesity* (aHRs = 1.13, 95% CIs: 0.99–1.29 for both outcomes). Because of the violation of the proportionality of hazards assumption in the overall follow-up period for *pure hyperglyceridemia*, we conducted the analyses for this particular outcome separately in the earlier and in the later time period (Supplemental Table 5). PC1 was associated with an elevated risk for *pure hyperglyceridemia* in the earlier (aHR = 1.59, 95% CIs: 1.02–2.49), but not in the later time period (aHR = 0.82, 95% CIs: 0.48–1.41).

PC2 was associated with elevated risks for a few conditions in the overall follow-up period, including *disorders of lipid metabolism* (aHR = 1.11, 95% CIs: 1.01–1.23) and its subcategory *other and unspecified hyperlipidemia* (aHR = 1.11, 95% CIs: 1.01–1.24), and *dysmetabolic syndrome X* (aHR = 1.74, 95% CIs: 1.19–2.54), now referred to as metabolic syndrome. After FDR-adjustment, only *dysmetabolic syndrome X* (p < 0.01; p_FDR_ < 0.20) was still statistically significant. There were also suggestions of elevated risks for *pure hypercholesterolemia* (aHR = 1.20, 95% CIs: 0.99–1.46), *overweight, obesity, and other hyperalimentation* (aHR = 1.10, 95% CIs: 0.99–1.24), and its subcategories of *overweight and obesity* (aHR = 1.11, 95% CIs: 0.99–1.24) and *overweight* (aHR = 1.14, 95% CI 0.99–1.31). The proportionality of hazards assumption was violated for two thyroid disease outcomes, *thyroid disorders combined* and *acquired hypothyroidism* during the overall follow-up period. PC2 was associated with reduced risks for both *thyroid disorders combined* (aHR = 0.77, 95% CI 0.47–1.25) and *acquired hypothyroidism* (aHR = 0.47, 95% CI 0.20–1.13) in the earlier time period and elevated risks for these two outcomes in the later time period (aHRs = 1.27 and 1.43, respectively), although none of the aHRs was statistically significant (Supplemental Table 5).

In the overall follow-up period, PC3 was associated with elevated risks for *disorders of lipid metabolism* (aHR = 1.11, 95% CIs: 1.00–1.23) and its subcategory *other and unspecified hyperlipidemia* (aHR = 1.13, 95% CIs: 1.01–1.26), and *overweight and obesity* (aHR = 1.13, 95% CIs: 1.00–1.27). After FDR-adjustment, this finding was not statistically significant.

PC4 was not significantly associated with risks for any of the endocrine and metabolic outcomes in the overall follow-up period. The proportionality of hazards assumption was violated for *acquired hypothyroidism* and *diabetes mellitus* in the overall follow-up period, however, period-specific analyses did not show any significant associations between PC4 and those two outcomes, although the number of cases of these outcomes was small (Supplemental Table 5).

Finally, during the overall study follow-up, PC5 was associated with elevated risks for *thyroid disorders combined* (aHR = 1.31, 95% CIs: 1.05–1.63), *acquired hypothyroidism* (aHR = 1.42, 95% CIs: 1.10–1.84)*,* and *obesity, unspecified* (aHR = 1.16, 95% CIs: 1.00–1.36). After FDR-adjustment, these findings were not statistically significant. Because of the violation of the proportionality of hazards assumption in the overall period for *gout* and *overweight*, the associations between PC5 and those two outcomes were separately examined in the earlier and the later time periods (Supplemental Table 5). The risk for *gout* was suggestively elevated only in the later time period (aHR = 1.45, 95% CI 0.99–2.12), while the risk for *overweight* was elevated only in the earlier time period (aHR = 1.22, 95% CI 1.03–1.44).

## Discussion

In this prospective cohort study of young, active duty USCG service members with military healthcare coverage designed for universal access, we found that different oil spill response stressor patterns were associated with increased risks for diagnoses of several neurological and endocrine/metabolic conditions during the approximately five years post-DWH response. Because our objective was to characterize multi-stressor response environment rather than external chemical exposures alone, we considered multiple deployment-related chemical, physical, and psychological stressors and characterized spill response patterns based on those stressors using a data reduction approach (i.e., PCA). We identified a five-component solution that explained nearly 80% of the total variance. The resulting components did not all represent complex mixtures of multiple variables. In particular, PCs 4 and 5 were dominated by single stressor dimensions (e.g., back pain, anxiety), indicating relatively independent aspects of the deployment experience. Accordingly, these components should be interpreted as stressor patterns rather than chemical mixtures. The five PCs were examined as exposures in relationship with various longer-term neurological and endocrine/metabolic outcomes in models adjusted for covariates and co-adjusted for each PC exposure, which were uncorrelated. PCs 1–3 were broadly characterized by higher chemical exposures (e.g., crude oil, vehicle exhaust, etc.) and generally associated with higher risk for conditions related to headaches and migraines. PCs 2 and 3 were additionally associated with disorders of lipid metabolism. PC4, characterized by high anxiety and modest fatigue, was associated with elevated risk of *abnormality of gait*. PC5, characterized by high back pain, was positively associated with mononeuritis, conditions related to lightheadedness and dizziness, *abnormality of gait*, thyroid disorders, and *obesity*. While FDR adjustments resulted in many of these findings no longer being statistically significant, our study aimed to assess patterns of risk, rather than to test specific hypotheses.

### Neurologic outcomes

To our knowledge, our study is the first to use this deployment-related stressor pattern approach in relation to longer-term outcomes following an oil spill cleanup. In the only other study that used a a similar approach to characterize DWH oil spill exposure patterns based on different types of spill-related stressors (i.e., self-reported physical, environmental, and economic exposures), Peres and colleagues observed that among women who resided in the proximity of the DWH spill, increased levels of both physical-environmental and economic stressors to the spill were associated with elevated reports of acute neurological symptoms (i.e., dizziness, headaches, blurry/distorted vision) up to eight months post-spill (Peres et al. 2016). Our findings of associations between stressor patterns characterized by physical and environmental (chemical) stressors and elevated risks for longer-term neurological conditions further support the results by Peres et al. (Peres et al. 2016). While the DWH disaster had a large economic impact on local responders and volunteers, this additional stressor should not have had a significant influence on our largely non-local, active duty military population, which deployed primarily from non-Gulf states (71%) and had a consistent source of income from the USCG.

In the present study, the first three PCs, characterized by different degrees of self-reported chemical exposures to crude oil, dispersants, and exhaust fumes, were generally associated with elevated risks for headache- and migraine-related conditions. Headaches have been associated with exposures to some crude oil and dispersant constituents, such as volatile organic compounds (VOCs), hydrogen sulfide, and 2-butoxyethanol, which may explain our findings. The associations between the first three PCs and elevated migraines/headaches risks are also in agreement with our previous findings of elevated risks for *headache* (aHR = 1.47, 95% CI 1.07–2.04), *other headache syndromes* (aHR = 1.83, 95% CI 1.03–3.25), *headaches/migraines combined* (aHR = 1.41, 95% CI 1.08–1.84), and *headaches/migraines combined excluding menstrual migraine and persistent migraine aura with cerebral infarction* (aHR = 1.39, 95% CI 1.06–1.81) among USCG responders who reported ever (vs. never) exposure to crude oil inhalation (Denic-Roberts et al. 2023). Additionally, risk estimates for headache and migraine conditions were higher in magnitude among responders reporting exposure to both crude oil and dispersants compared to those reporting neither exposure than among responders reporting exposure to crude oil only (vs. neither exposure), which is in line with our current findings for the PC2 that was characterized by a high crude oil and dispersant exposure pattern (Denic-Roberts et al. 2023). In the present analysis, PC2 was also associated with increased risk for *migraine with aura* (aHR = 1.54, 95% CI 1.14–2.07), while the association between the crude oil inhalation exposure and this outcome was suggestive (aHR = 2.22, 95% CI 0.98–5.01) in our prior study (Denic-Roberts et al. 2023).

In addition to a moderate exhaust inhalation exposure and a modest crude oil exposure via skin contact, the stressor pattern of PC1 was also characterized by high levels of working outdoors and heavy PPE use, two factors that could have contributed to heat stress. While acute health effects of heat stress are well documented even among the DWH responders, chronic health effects related to heat stress, especially in occupational settings, are less understood. Along with the moderate exhaust inhalation exposure and the modest crude oil inhalation exposure, the stressor pattern of PC3 was also characterized by a high level of fatigue, a condition often interconnected with neurological disorders, including headaches and migraines. We can also not rule out a possibility that fatigue was an early indicator of undiagnosed neurological conditions.

In the present study, PC2 (high crude oil and dispersant exposure pattern) was also associated with an increased risk for an inflammatory nerve condition *mononeuritis of upper limb and mononeuritis multiplex*. Both self-reported crude oil inhalation exposure (aHR = 1.71, 95% CI 1.04–2.83) and a combination of exposure to crude oil and dispersants (aHR = 2.71, 95% CI 1.34–5.49) were associated with elevated *mononeuritis of upper limb and mononeuritis multiplex* risk in our previous study (Denic-Roberts et al. 2023). Given that *mononeuritis of upper limb and mononeuritis multiplex* is a type of peripheral neuropathy and that crude oil constituents (e.g., VOCs) can affect the PNS, it is reasonable that we observed this association in both studies.

The stressor pattern dominated by high anxiety and modest fatigue levels (PC4) was associated with an increased risk for *abnormality of gait*. While anxiety symptoms have been related to a decline in self-reported functioning, including impaired walking, in older adults, our finding was unexpected in a cohort of young, active duty USCG service members. This association could have been a result of statistical chance (type I error), given the low number of responders with this diagnosis (n = 35) and should therefore be interpreted with caution and investigated in other studies.

We found that the stressor pattern dominated by high levels of back pain (PC5), was associated with an increased risk for *mononeuritis of lower limb*, an inflammatory nerve condition that typically involves symptoms of pain, numbness, and tingling in a single-nerve distribution. This association may indicate inflammatory processes not isolated to a single nerve. Since back pain can impede one’s mobility, it is understandable that it would be associated with *abnormality of gait*. It is not clear why PC5 was also associated with *syncope and collapse* and *dizziness and giddiness*.

### Endocrine and metabolic outcomes

Sub-clinical endocrine outcomes have been previously studied in a handful of investigations following the 2002 *Prestige* oil spill off the coast of Northwestern Spain. Those investigators observed acute and longer-term alterations in biomarkers of potential endocrine toxicity (i.e., plasma prolactin and cortisol levels) among exposed oil spill cleanup workers and volunteers. We could not compare our findings to those from the *Prestige* spill because we did not have laboratory values for the USCG DWH responders, and ICD-based conditions related to alterations in prolactin and cortisol (i.e., hyperprolactinemia or galactorrhea not associated with childbirth) were too rare to study in our cohort.

The GuLF Study investigators conducted a prospective study among 2,660 DWH oil spill cleanup workers to investigate the relationship between an estimated total hydrocarbon (THC) exposure and risk of type 2 diabetes mellitus (T2DM) up to six years post-spill. Their incident T2DM definition required either a self-report of post-DWH T2DM physician diagnosis, a self-report of antidiabetic medication use, or a hemoglobin A1c level of ≥ 6.5% measured during a clinical exam conducted several years after the spill. Overall, the association between increasing THC exposure and incident T2DM risk was not significant (*p*-trend = 0.22); however, there was a significant trend in a restricted sample of 843 overweight individuals (Relative Risk [RR]_0.30–0.99 vs. <0.30 ppm_ = 0.99, 95% CI 0.37–2.69, RR_1.00–2.99 vs. <0.30 ppm_ = 1.46, 95% CI 0.54–3.92, RR_≥3.00 vs. <0.30 ppm_ = 2.11, 95% CI 0.78–5.74, p-trend = 0.03). In our study, the number of incident DM cases was small (n = 20) and the associations between the first three PCs (stressor patterns characterized by chemical exposures, including to hydrocarbons) and incident DM were largely null (aHRs = 0.91, 0.81, and 0.88 for PC1, PC2, and PC3, respectively). Because of the small number of DM cases in our population, restricting our sample to overweight individuals for comparison to the GuLF Study findings was not feasible.

In our study, both PC2 (high crude oil via inhalation and direct skin contact/high dispersants stressor pattern) and PC3 (high fatigue/moderate exhaust and crude oil inhalation stressor pattern) were associated with an increased risk for dyslipidemia, one of the major risk factors for T2DM. This finding is in agreement with our previous study among the USCG DWH responders, where we observed an elevated risk for *disorders of lipid metabolism* in relation to self-reported exposures to crude oil inhalation, to crude oil via direct skin contact and to a combination of crude oil and dispersant exposure*.* These findings are also supported by evidence from in vitro studies that demonstrated the endocrine disrupting potential of polycyclic aromatic hydrocarbons (PAHs), a constituent of crude oil and exhaust fumes. PC2 and PC3 were also marginally associated with increased *overweight and obesity* diagnoses, which strengthens the evidence from our previous study of associations between self-reported exposures to crude oil inhalation, crude oil skin contact, and both crude oil and dispersants and increased risk for overweight and obesity-related conditions. Chemical exposures to PAHs and to dioctyl sodium sulfosuccinate (DOSS), a constituent in both COREXIT dispersants, have been associated with disruption of endocrine function in vivo and in vitro through various mechanisms, including alterations in steroidogenesis and lipogenesis, and significant changes in peroxisome proliferator activated receptor gamma (*PPARγ*) gene expression (a molecular target for obesity), further supporting the biological plausibility of our findings. The association we observed between the PC2 stressor pattern and elevated risk of metabolic syndrome, a condition that is partially defined by excess body fat and abnormal cholesterol, is also supported by the endocrine-disrupting potential of PAHs and DOSS.

PC5, characterized by the high back pain stressor pattern, was associated with increased risks for thyroid disorders, including *acquired hypothyroidism*, and *obesity*. We are not aware that back pain is an independent risk factor for thyroid-related disorders. However, since back pain affects mobility, reduced physical activity or increased body mass index could be potential mediators in this relationship given that both have been associated with alterations in thyroid hormone levels. However, we cannot rule out the possibility that thyroid disorders preceded back pain but were undiagnosed. Back pain leading to reduced mobility may also help explain the association between PC5 and elevated risk for *obesity*.

### Strengths and limitations

This study has several strengths. We used a deployment-related stressor pattern approach to characterize spill response exposure patterns using several types of stressors, including chemical, physical, and psychological. This approach allowed us to model a realistic disaster response exposure scenario of multiple occupational stressors occurring simultaneously. We obtained longer-term health outcomes from an objective military health encounter database with comprehensive coverage of inpatient and outpatient visits occurring in both military and civilian facilities, reducing potential for outcome misclassification. Because we had access to health records from before the DWH spill response, we were able to exclude pre-existing (prevalent) conditions and, therefore, assess incident neurological, endocrine, and metabolic health outcomes. Since our study population was comprised of young active duty service members who were healthy and fit for deployment, the likelihood of existing co-morbidities at the time of the DWH response was low.

Our findings should be interpreted considering a few limitations. First, spill response stressors were based on self-report, which may be subject to differential recall or recall errors. Responders who experienced adverse symptoms or diagnoses prior to completion of the post-deployment survey may be more likely to provide a more complete report of exposures, resulting in a biased estimate away from the null (Neugebauer & Ng 1990). However, responders completed the post-deployment survey used for this analysis fairly shortly following the deployment (a median of 153 days post-deployment) (Rusiecki et al. 2017), minimizing potential for recall errors due to time elapsed. Additionally, measuring oil spill exposures by self-report is challenging because of a lack of validated questionnaires. We characterized spill stressor patterns using PCA, a data-driven approach that largely relies on interpretability of PCs. Nevertheless, we used expert judgement, along with previously established criteria (e.g., 80% of total variance being explained by the PCs) to guide our selection process. Some of our associations could be statistically significant due to chance since we carried out multiple comparisons across five PCs and a range of outcomes. However, given the paucity of research examining longer-term neurological and endocrine/metabolic outcomes following an oil spill response, our main goals were to 1) assess patterns of risk, rather than to test any specific hypotheses, and 2) expand upon our previous findings using a realistic multi-stressor framework.

Although we used objectively ascertained military health encounter records to define our study outcomes, ICD coding can be subject to classification inaccuracies (O’Malley et al. 2005). However, for identification of some of the outcomes that we evaluated (e.g., dyslipidemia, abnormal blood glucose, and diabetes mellitus) blood testing is required in the MHS (O’Donnell et al. 2018). On the other hand, some of the other outcomes that we examined, such as obesity, are prone to high levels of underdiagnosis (Kapoor et al. 2020). Nevertheless, with proper interpretation, ICD coding is generally a reliable indicator of medical diagnoses, and has been used widely in observational studies (O’Malley et al. 2005) and military surveillance (O’Donnell et al. 2018). To increase the diagnostic accuracy of ICD coding in our own study, the incident case definitions required at least one inpatient or two outpatient medical encounters. It should be noted that non-differential misclassification of diagnosis could bias HRs towards the null; therefore, results in this analysis could be an underestimation of actual effects. Given that active duty military members have equal access to health care and are required to complete annual health assessments, the responders in our cohort should have had an equal opportunity to be diagnosed with any conditions, regardless of their DWH response stressor pattern. Despite equal access to care, health-seeking behavior may vary among military personnel. For example, a responder with high anxiety, such as responders in PC4, may experience social anxiety and be less likely to visit a doctor. This could manifest in detection bias. Lastly, the generalizability of our findings to oil spill responders from the general population may be limited because our cohort consisted of active duty military members who were young and predominantly male.

## Conclusions

Overall, our findings suggest that oil spill response stressor patterns experienced by the USCG responders to the DWH oil spill were adversely associated with neurological and endocrine/metabolic health outcomes up to five years following the spill cleanup, with associations varying by PC. PCs broadly characterized by higher chemical exposures (e.g., crude oil, vehicle exhaust, etc.) were generally associated with higher risk for conditions related to headaches and migraines; two out of three of these PCs were also associated with increased risk for *disorders of lipid metabolism*. The PC characterized by high anxiety and modest fatigue was associated with elevated risk of *abnormality of gait*. The PC characterized by high back pain was associated with elevated risk of mononeuritis, conditions related to lightheadedness and dizziness, *abnormality of gait*, thyroid disorders, and *obesity*. These adverse health outcomes may in turn adversely impact the readiness of the USCG service members. To our knowledge, this is the first study to examine longer-term health outcomes in association with different types of spill response-related stressors using a deployment-related stressor pattern approach. Our findings highlight the need for future investigations to examine long-term health effects associated with the complex occupational exposure scenarios of disaster cleanup.

## Data Availability

The datasets used and/or analyzed during the current study are available from the corresponding author on reasonable request.

## Abbreviations

aHR: Adjusted hazard ratios
BTEX: Benzene, toluene, ethylbenzene, and xylenes
CI: Confidence interval
DOSS: Dioctyl sodium sulfosuccinate
DWH: Deepwater horizon
DWH-CG: Deepwater Horizon Coast Guard
FDR: False discovery rate
GuLF: Gulf Long-term Follow-up
ICD: International Classification of Diseases
IRB: Institutional Review Board
LCA: Latent class analysis
MDR: Military Health System Data Repository
MHS: Military Health System
NIOSH: National Institute for Occupational Safety and Health
PAH: Polycyclic aromatic hydrocarbons
PC: Principal component
PCA: Principal component analysis
PNS: Peripheral nervous system
PPAR: Peroxisome proliferator activated receptor
PPE: Personal protective equipment
RR: Relative risk
THC: Total hydrocarbon
T2DM: Type 2 diabetes mellitus
USCG: United States Coast Guard
USU: Uniformed Services University
VOC: Volatile organic compounds
WQS: Weighted quantile sum
